# Markers of intestinal inflammation in patients with ankylosing spondylitis: a pilot study

**DOI:** 10.1186/ar4106

**Published:** 2012-11-29

**Authors:** Franziska G Matzkies, Stephan R Targan, Dror Berel, Carol J Landers, John D Reveille, Dermot PB McGovern, Michael H Weisman

**Affiliations:** 1 Department of Medicine, Division of Rheumatology, University of California, San Francisco, 533 Parnassus Avenue, Room U384/ Box 0633, San Francisco, CA 94143, USA; 2 Inflammatory Bowel and Immunobiology Research Institute, Cedars-Sinai Medical Center, 8700 Beverly Boulevard, Los Angeles, CA 90048, USA; 3 Division of Rheumatology, University of Texas, 6410 Fannin Street, Houston, TX 77030, USA; 4 Medical Genetics Institute, Cedars-Sinai Medical Center, 8700 Beverly Boulevard, Los Angeles, CA 90048, USA; 5 Division of Rheumatology, Cedars-Sinai Medical Center, 8700 Beverly Boulevard, Becker B-131, Los Angeles, CA 90048, USA

## Abstract

**Introduction:**

Inflammatory bowel disease (IBD) and ankylosing spondylitis (AS) are similar chronic inflammatory diseases whose definitive etiology is unknown. Following recent clinical and genetic evidence supporting an intertwined pathogenic relationship, we conducted a pilot study to measure fecal calprotectin (fCAL) and IBD-related serologies in AS patients.

**Methods:**

Consecutive AS patients were recruited from a long-term prospectively collected longitudinal AS cohort at Cedars-Sinai Medical Center. Controls were recruited from Cedars-Sinai Medical Center employees or spouses of patients with AS. Sera were tested by ELISA for IBD-associated serologies (antineutrophil cytoplasmic antibodies (ANCA), anti-*Saccharomyces cerevisiae *antibody IgG and IgA, anti-I2, anti-OmpC, and anti-CBir1). The Bath Ankylosing Spondylitis Disease Activity Index, the Bath Ankylosing Spondylitis Functional Index, and the Bath Ankylosing Spondylitis Radiology Index were completed for AS patients.

**Results:**

A total of 81 subjects (39 AS patients and 42 controls) were included for analysis. The average age of AS patients was 47 years and the average disease duration was 22 years. AS patients were predominantly male; 76% were HLA-B27-positive. Median fCAL levels were 42 μg/g and 17 μg/g in the AS group and controls, respectively (*P *< 0.001). When using the manufacturer's recommended cutoff value for positivity of 50 μg/g, stool samples of 41% of AS patients and 10% of controls were positive for fCAL (*P *= 0.0016). With the exception of ANCA, there were no significant differences in antibody levels between patients and controls. Median ANCA was 6.9 ELISA units in AS patients and 4.3 ELISA units in the controls. Among AS patients stratified by fCAL level, there were statistically significant differences between patients and controls for multiple IBD-associated antibodies.

**Conclusion:**

Calprotectin levels were elevated in 41% of patients with AS with a cutoff value for positivity of 50 μg/g. fCAL-positive AS patients displayed higher medians of most IBD-specific antibodies when compared with healthy controls or fCAL-negative AS patients. Further studies are needed to determine whether fCAL can be used to identify and characterize a subgroup of AS patients whose disease might be driven by subclinical bowel inflammation.

## Introduction

Inflammatory bowel disease (IBD) and ankylosing spondylitis (AS) are similar chronic inflammatory diseases whose definitive etiology is unknown. Although both appear to be distinct and well-defined phenotypes, there is increasing clinical and genetic evidence supporting an intertwined pathogenic relationship [1]. Clinically, 5 to 10% of all patients with AS have concurrent IBD [2]. In patients with AS without specific gastrointestinal complaints, macroscopic gut inflammation resembling Crohn's disease has been observed in 25 to 50% of all patients by colonoscopy [3]. Histological analyses of gut biopsies in AS patients have observed evidence of microscopic gut inflammation even more frequently with a prevalence of 50 to 60% [4-7]. Alternatively, sacroiliac changes similar to those seen in AS have been noted in 10 to 20% of patients with the primary diagnosis of IBD and 7 to 12% of IBD patients carry the concomitant diagnosis of AS [8].

The genetic susceptibility risk is high in both conditions [9-13] and recent studies with large and well-characterized cohorts support an important genetic overlap between AS and IBD. The Icelandic genealogy database has shown that AS and IBD have a strong elevated cross-risk ratio in first-degree and second-degree relatives [14]. HLA-B27 contributes approximately one-half of the genetic risk for AS, and the prevalence of the HLA-B27 positivity in AS approaches 90% [15]. In studies of patients with IBD with associated spondylitis and sacroiliitis, the prevalence of HLA-B27 varies from 25 to 78% depending on the study [16]. In the absence of sacroiliitis, the prevalence of HLA-B27 in IBD is not different from that in healthy controls (reviewed in [17]). Recently published large genome-wide association studies have identified multiple non-major histocompatibility complex susceptibility loci that provide additional evidence for a genetic overlap between Crohn's disease and AS. These loci include I*L23R, STAT3, IL-12B, TNFSF15 *and the intergenic region at chr1q32. The chr1q32 is located close to the *KIF21B*, a gene that encodes for kinesin motor proteins [18]. *IL-23R, STAT3 *and *IL-12p40 *all play an important role in the Th17 inflammatory pathway.

Mucosal dysregulation may be an important pathway linking genetic susceptibility to environmental triggers in both conditions. Patients with IBD and AS both have increased intestinal permeability as shown by the ^31^Cr-ethylenediamine tetraacetic acid resorption test [19]. Loss of tolerance to normal bowel flora as evidenced by an increase in circulating antibodies to certain bacterial antigens has been observed in IBD, including anti-*Saccharomyces cerevisiae *mannan antibodies (ASCA) [20], anti-*Escherichia coli *outer-membrane porin C (OmpC) and anti-CBir1 flagellin AB [21]. The same IBD-associated serologic markers present at a greater frequency and at a higher titer in AS patients compared with healthy controls, indicating a potential loss of tolerance to commensal bacteria similar to that observed in IBD patients [22-25].

Calprotectin is a neutrophil-derived protein that can be quantified in the feces and has become established as a marker of whole gut inflammation. The increase of fecal calprotectin (fCAL) seen in the stool of IBD patients is a direct result of increased neutrophil migration into the gut lumen across inflamed mucosa (as reviewed in [26]). In subjects with IBD, levels of fCAL correlate with endoscopic and histological degree of bowel inflammation [27,28]. Furthermore, fCAL has been successfully shown to predict relapses and detect pouchitis in patients with IBD and to consistently differentiate IBD from irritable bowel syndrome.

Although IBD is typically diagnosed by direct observation (colonoscopy) and confirmed with biopsy and histology, finding alternative ways to assess subclinical inflammation in a large cohort of patients would be beneficial for epidemiologic studies and continued long-term multi-disciplinary investigations into the relationship between these two diseases. To further address the question of subclinical bowel inflammation in AS patients, we measured fCAL combined with IBD-related serologies in this pilot exploratory study.

## Materials and methods

### Patient selection

Consecutive AS patients were recruited from our long-term prospectively collected longitudinal AS cohort at Cedars-Sinai Medical Center (CSMC) [29]. All patients were 18 years of age or older, supplied written consent, and met the modified New York criteria for AS [30] at the time of enrollment. Healthy controls were recruited from CSMC employees or spouses of patients with AS. Healthy controls were 18 years of age or older, supplied written consent, and were free from any history of rheumatic disease or IBD. AS patients and controls with a personal or family history of IBD were excluded from the study. AS patients undergoing treatment with mAbs against TNFα were either not invited to participate or were later excluded. Patients undergoing treatment with a soluble TNFα receptor (Enbrel; Etanercept; manufactured by Immunex Corporation, Thousand Oaks, CA 91320. Marketed by Amgen Inc., Thousand Oaks, CA 91320 and Pfizer Inc. USA) were allowed to participate. Patients and controls using NSAIDs within 2 days of the stool collection were excluded. The study was approved by the CSMC Institutional Review Board.

### Laboratory methods

#### Stool samples

All participants collected stool samples at home or provided a sample during study visits. Stool samples were handled according to manufacturer recommendations (Genova Diagnostics, Asheville, NC, USA) and were frozen within 48 hours at -20°C. Samples were coded and sent to Genova Diagnostics for analysis.

#### Serum samples

All serum samples were collected during the study visit at CSMC. Sera were tested by ELISA for the IBD-related serologies: antineutrophil cytoplasmic antibodies (ANCA), ASCA IgG and IgA, anti-I2, anti-OmpC, and anti-CBir1. Serologies were all estimated by ELISA at the same time in an effort to decrease variance. The basic laboratory methods for the determination of each serology have been described previously [31,32]. The results of each assay are expressed in ELISA units.

### Additional measures

The Bath Ankylosing Spondylitis Disease Activity Index (BASDAI), the Bath Ankylosing Spondylitis Functional Index (BASFI), and the Bath Ankylosing Spondylitis Radiology Index (BASRI) instruments were completed for AS patients in addition to an assessment of peripheral arthritis.

### Statistical methods

ASCA results were log-transformed prior to quartile distribution. The mean age and disease duration between fCAL-positive and fCAL-negative AS patients were analyzed using Student's *t *test. The exact Fisher two-sided test was performed to compare positivity rates for fCAL-positive versus fCAL-negative AS patients. The Mann-Whitney U test was performed to compare the median values for fCAL, BASDAI score, BASRI score, and quantitative antibody levels between AS patients and healthy controls.

## Results

Calprotectin samples were initially collected from 47 AS patients and 42 healthy controls. Seven AS patients were excluded from further analysis for use of prohibited NSAIDs within 48 hours of the stool collection, and one AS patient was excluded for treatment with TNFα mAb prior to the study visit, thereby yielding a total of 81 study participants with data suitable for analysis. Demographic data for AS patients and controls are reported in Table 1. The average age of the AS patients was 47 years and the average disease duration was 22 years. AS patients were predominantly male and 76% were HLA-B27-positive.

**Table 1:** Demographic data for ankylosing spondylitis patients and healthy controls

	Ankylosing spondylitis patients (*n *= 39)	Healthy controls (*n *= 42)
Age (years)	47 (20 to 85)	39 (27 to 70)
Male	82	52
HLA-B27	76	Not available
Disease duration (years)	22 (2 to 62)	Not applicable
Ethnicity		
Caucasian	77	54
Asian	8	36
Hispanic	15	10

Data presented as mean (range) or percentage.

Positivity rates and fCAL levels are reported in Table 2. The median fCAL levels were 42 μg/g and 17 μg/g in the AS group and controls, respectively (*P *= 0.0002). When using the manufacturer's recommended cutoff value for positivity of 50 μg/g, stool samples of 41% of AS patients and 10% of controls were positive for fCAL (*P *= 0.0016).

**Table 2:** Positivity rates and level of fecal calprotectin in ankylosing spondylitis patients and healthy controls

	Ankylosing spondylitis patients (*n *= 39)	Healthy controls (*n *= 42)	*P *value
Fecal calprotectin (μg/g)	42 (16 to 1,085)	17 (16 to 104)	0.0002
Fecal calprotectin positive (positive > 50 μg/g)	16 (41%)	4 (10%)	0.0016

Data presented as mean (range) or number (percentage).

**Figure 1:**
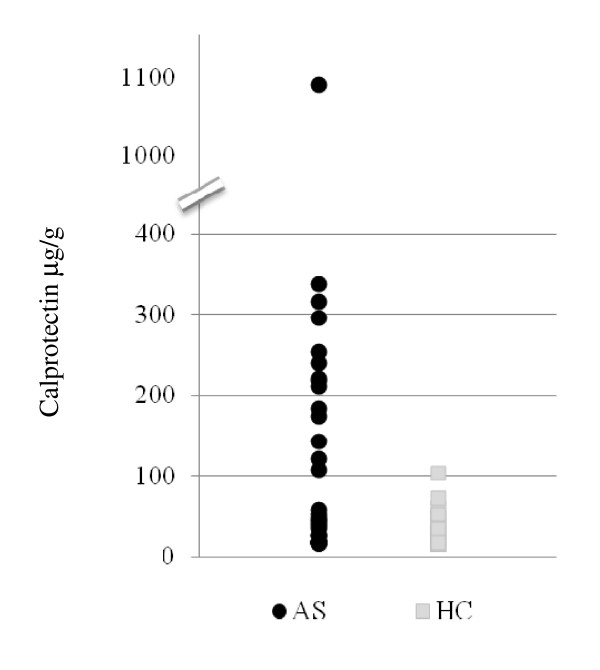
**Fecal calprotectin levels in healthy controls and patients with ankylosing spondylitis**. Absolute values of fecal calprotectin (fCAL) are shown in μg/g (*y *axis). Each square represents the fCAL level of an individual healthy control (HC) and each circle represents the fCAL of an individual patient with ankylosing spondylitis (AS). The highest measured level of fCAL in the control group was 104 μg/dl, while the highest observed level in the AS group was 1,085 μg/g.

Calprotectin levels for AS patients and controls are displayed in Figure 1. In general, the fCal levels of AS patients are higher than the fCAL levels of controls. The highest measured level of fCAL in the control group was 104 μg/g, while the highest observed level in the AS group was 1,085 μg/g. Demographic and clinical parameters of AS subjects stratified by fCAL positivity are shown in Tables 3 and 4. There were no significant differences between the fCAL-positive and fCAL-negative AS patients with regards to age, gender, ethnicity, disease duration and HLA-B27 positivity. There was also no significant difference in NSAID use or Enbrel use (Table 4). Although the median BASFI in the fCAL-positive group was 41.6, compared with 17 in the fCAL-negative group, this association was not statistically significant. There was no significant difference for the BASRI or the occurrence of peripheral arthritis between these two groups of AS patients (Table 4).

**Table 3:** Demographic data for ankylosing spondylitis patients stratified by fecal calprotectin positivity

	Fecal calprotectin cutoff 50 μg/g		
	
	Fecal calprotectin positive (*n *= 16)	Fecal calprotectin negative (*n *= 23)	*P *value
Mean age (years)	45	49	0.48
Male, *n *(%)	14 (88)	18 (78)	0.68
HLA-B27-positive, *n *(%)	11 (69)	18 (82)	0.71
Mean disease duration (years)	22	22	0.96
Ethnicity (%)			
Caucasian	8	74	0.71
Asian	0	13	0.26
Hispanic	19	13	0.67

**Table 4:** Clinical data for ankylosing spondylitis patients in stratified by fecal calprotectin positivity

	Fecal calprotectin cutoff 50 μg/g		
	
	Fecal calprotectin positive (*n *= 16)	Fecal calprotectin negative (*n *= 23)	*P *value
NSAID use	6 (38%)	10 (43%)	0.75
Enbrel use	9 (56%)	8 (35%)	0.20
Patients waking up at night for bowel movement	3 (19%)	3 (13%)	0.67
BASDAI score	3.0 (0.1 to 7.75)	1.8 (0.03 to 7.66)	0.16
BASRI score	6.0 (2 to 13.5)	5.0 (2 to 11)	0.64
BASFI score	41.6 (0.4 to 92.5)	17.1 (0 to 72.1)	0.09
Erythrocyte sedimentation rate	8.5 (0 to 62)	17 (2 to 64)	0.75
C-reactive protein	0.44 (0.03 to 5.7)	0.57 (0.02 to 4.84)	0.58
Patients with history of uveitis	7 (44%)	10 (43%)	1.0
Patients with history of peripheral arthritis in the knee	4 (25%)	5 (22%)	1.0
Patients with history of peripheral arthritis in the ankle	5 (31%)	4 (17%)	0.44

Data presented as median (range) or number (percentage). BASDAI, Bath ankylosing spondylitis disease activity index; BASFI, Bath ankylosing spondylitis functional index; BASRI, Bath ankylosing spondylitis radiology index.

With the exception of ANCA, there were no significant differences in antibody levels between patients and controls (Table 5). The median ANCA value was 6.9 ELISA units in AS patients and 4.3 ELISA units in the controls (*P *= 0.003). At the fCAL threshold of 50 μg/g, there were statistically significant differences between AS fCAL-positive patients and controls (*P *< 0.05) for ANCA, ASCA IgG, and anti-CBir1 antibodies (9.2 vs. 4.3, 11.5 vs. 6.7, and 27.2 vs. 15.9, respectively). There was no statistically significant difference between the AS fCAL-negative patients compared with the healthy controls (Table 6).

**Table 5:** Serologies of ankylosing spondylitis patients and healthy controls

	Healthy controls (all) versus AS patients (all)		
	
	Median healthy controls (all)	Median AS patients (all)	*P *value
IgA ASCA (ELISA units)	0.4	0.5	0.737
IgG ASCA (ELISA units)	6.7	6.6	0.597
I2 (ELISA units)	5.7	6	0.685
OmpC (ELISA units)	5.7	6.4	0.473
Cbir1 Fla (ELISA units)	15.9	19.8	0.223
ANCA (ELISA units)	4.3	6.9	0.003

ANCA, antineutrophil cytoplasmic antibodies; AS, ankylosing spondylitis; ASCA, anti-*Saccharomyces cerevisiae *antibody; CBir1, anti-flagellin; OmpC, anti-*Escherichia coli *outer membrane porin C.

**Table 6:** Serologies of ankylosing spondylitis patients and healthy controls stratified by fecal calprotectin

	Healthy controls (all) compared with AS patients (fCAL cutoff 50 μg/g)				
	
	Median HC	Median AS fCAL-negative	*P *value (HC versus fCAL-negative)	Median AS fCAL-positive	*P *value (HC versus fCAL-positive)
IgA ASCA (ELISA units)	0.4	0.2	0.478	1.9	0.110
IgG ASCA (ELISA units)	6.7	6.2	0.344	11.5	0.023
I2 (ELISA units)	5.7	6.8	0.394	4	0.703
OmpC (ELISA units)	5.7	6.1	0.904	8.4	0.240
Cbir1 Fla (ELISA units)	15.9	16.2	0.659	27.2	0.003
ANCA (ELISA units)	4.3	5	0.240	9.2	< 0.001

ANCA, antineutrophil cytoplasmic antibodies; AS, ankylosing spondylitis; ASCA, anti-*Saccharomyces cerevisiae *antibody; CBir1, anti-flagellin; fCAL, fecal calprotectin; HC, healthy controls; OmpC, anti-*Escherichia coli *outer membrane porin C.

When fCAL-positive AS patients were compared with fCAL-negative AS patients at the 50 μg/g threshold, significant differences were found in IgA ASCA levels (*P *= 0.05), IgG ASCA levels (*P *= 0.003), and Cbir1 (*P *= 0.002; Table 7). A trend towards significant association was seen in ANCA levels (*P *= 0.06; Table 7).

**Table 7:** Serologies of patients with ankylosing spondylitis stratified by fecal calprotectin

	AS fCAL-positive versus fCAL-negative (cutoff 50 μg/g)		
	
	Median AS fCAL-positive	Median AS fCAL-negative	*P *value
IgA ASCA (ELISA units)	1.9	0.2	0.05
IgG ASCA (ELISA units)	11.5	6.2	0.003
I2 (ELISA units)	4	6.8	0.36
OmpC (ELISA units)	8.4	6.1	0.34
Cbir1 Fla (ELISA units)	27.2	16.2	0.002
ANCA (ELISA units)	9.2	5	0.06

ANCA, antineutrophil cytoplasmic antibodies; AS, ankylosing spondylitis; ASCA, anti-*Saccharomyces cerevisiae *antibody; CBir1, anti-flagellin; fCAL, fecal calprotectin; OmpC, anti-*Escherichia coli *outer membrane porin C.

## Discussion

In this exploratory study, we report a higher prevalence and increased levels of fCAL positivity as well as an association with the IBD serological markers ANCA, ASCA, anti-CBir1, anti-I2 and anti-OmpC antibodies in a well-characterized cohort of AS patients. The fCAL-positive AS patients in our study displayed higher medians of most IBD-specific antibodies when compared with healthy controls or fCAL-negative AS patients. Our finding of 41% of patients with fCAL > 50 μg/g is considerably lower than the 68% of patients with fCAL > 50 mg/kg found by Klingberg and colleagues [33]. However, the latter group contained AS subjects taking all of the usual clinically indicated medications, including NSAIDs and anti-TNF agents. In addition, in a small study of enthesitis-related arthritis in children - a condition felt to be a form of pediatric spondyloarthritis that is typically HLA B-29-positive - fCAL was shown to be a distinguishing feature for this form of juvenile idiopathic arthritis compared with other pediatric rheumatologic conditions and controls [34].

The relationship between AS and IBD has been under study in recent years. Increased gut permeability and inflammation in AS has been observed in multiple studies; however, the range of gut prevalence varies by the type of method used to assess inflammation. Invasive methods such as colonoscopies and biopsies have shown macroscopic and microscopic gut inflammation in 25 to 60% of AS patients while noninvasive observational studies report clinical overt IBD in 5 to 10% of AS patients (as reviewed in [35]). Our study is intended to evaluate a practical approach for further studies that will bridge the gap between invasive methodologies to determine prevalence of gut inflammation in AS and the less sensitive methods utilizing clinical histories and symptoms.

Although attempts have been made in the recent past to utilize biomarkers of gut inflammation in similar investigations such as ours, many of these studies have not controlled adequately for medications that can influence gut inflammation such as TNFα inhibitors or NSAIDs. We attempted to address this potential limitation using strict inclusion and exclusion criteria. mAbs against TNFα (infliximab, adalimumab and certolizumab pegol) have proven efficacy in IBD and are US Food and Drug Administration approved to treat IBD [36-39]. Etanercept, a TNFα receptor fusion protein, has not been shown to be efficacious in IBD but is widely used for AS [40]. We limited our patient study population to allow etanercept only. Further, NSAIDs have been shown to increase gut permeability and inflammation in healthy users [41]. The literature is unclear regarding how long the effects on the intestinal mucosa caused by NSAIDs will last. The manufacturer of the fCAL assay used in this study (Genova Diagnostics) recommends avoiding NSAIDs within 48 hours of collection. Adhering to this recommendation, we observed 41% of AS patients to display positive fCAL levels (fCAL > 50 μg/g). When all AS patients that used NSAIDS on a regular basis were excluded, the percentage of fCAL-positive patients changed only marginally (data not shown).

The increase of fCAL seen in the stool of IBD patients has been shown to be a direct result of increased neutrophil migration into the gut lumen across inflamed mucosa [26], and in subjects with IBD the levels of fCAL correlate with the endoscopic and histological degree of gut inflammation in adults and children [27]. A recent meta-analysis reported a sensitivity of 0.95 and a specificity of 0.91 for fCAL to diagnose IBD in adults. The diagnostic precision of fCAL had a better accuracy at a cutoff level of 100 μg/g versus 50 μg/g [42].

Interestingly, BASFI and BASDAI scores are higher for fCAL-positive patients compared with fCAL-negative AS patients. This finding might suggest more disease activity and functional disability in the group of patients with subclinical inflammation of the gut. Prior studies have found higher BASFI scores in ASCA-positive AS patients, confirming our findings [23]. There are two possible explanations for this observation. First, subclinical gut inflammation might lead to increased gut permeability and prolonged antigen exposure, triggering more severe disease as evidenced by higher BASDAI scores and more functional disability as evidenced by higher BASFI scores. The second possibility is that patients with higher BASDAI and BASFI scores have higher levels of gut inflammation due to more severe disease. Our sample numbers are too small to make any of these distinctions, however, and further studies are needed to confirm this interesting finding.

Analysis of serologies revealed that there were no significant differences between controls and fCAL-negative AS patients. However, when fCAL-positive AS patients were compared with fCAL-negative AS patients and controls, a significantly higher median was found for some of the IBD-specific antibodies. Calprotectin-positive AS patients compared with controls have significantly higher mean values for antibodies against OmpC, CBir1, and ANCAs, ASCA IgG and IgA. When fCAL-positive AS patients are compared with fCAL-negative AS patients, these findings are confirmed for antibodies against CBir1 and ASCA IgG and IgA, but not for antibodies against ANCA, OmpC or I2 (although trends towards significant association are seen with ANCA and OmpC). These results suggest that the fCAL-positive AS patient might have prolonged antigenic exposure to gut antigens as evidenced by the development of serologic memory. In addition, results in fCAL-positive AS patients suggest the importance of a potential bacterial trigger that could provide a link to new genetic discoveries. Our most recent findings that polymorphisms of ERAP1, which encode an endoplasmic reticulum aminopeptidase involved in peptide trimming before HLA class I presentation, only affects AS risk in HLA-B27-positive individuals provides strong evidence that HLA-B27 operates in AS through a mechanism involving aberrant processing of antigenic peptides [43].

There are important limitations to interpreting the results from this study. First, our sample size is small and a larger study is required to more precisely determine the prevalence of subclinical bowel inflammation in our own patient cohort. Our study is cross-sectional; longitudinal studies will be needed to determine the clinical significance of fCAL and IBD-specific serologies in AS. Second, although our patients were not taking NSAIDs for at least 48 hours prior to the study, there could be a carryover effect or patients may not have been compliant with the instructions. Third, we do not have concurrent colonoscopies or capsule studies of our AS patients that might confirm whether fCAL positivity truly correlates with bowel inflammation. However, fCAL testing has an excellent specificity and sensitivity in distinguishing IBD from non-IBD. Further studies of these relationships will serve to place patients in a clinical context and to provide associations with clinical characteristics, disease severity, and treatment response. Our preliminary data suggest that fCAL-positive AS patients have more active disease, a finding with significant clinical implications if verified in other studies.

## Conclusion

In summary, fCAL, a fecal marker for whole gut inflammation, as well as serological antibodies that have been well studied and characterized in IBD are found in a higher percentage of AS patients followed at our institution compared with healthy controls. Further studies, including more patients on a variety of medications, and longitudinal observations will be needed to prospectively determine whether fCAL can be used to identify and characterize a subgroup of AS patients whose disease might be driven by subclinical bowel inflammation.

## Abbreviations

ANCA: antineutrophil cytoplasmic antibodies; AS: ankylosing spondylitis; ASCA: anti-*Saccharomyces cerevisiae *antibody; BASDAI: Bath ankylosing spondylitis disease activity index; BASFI: Bath ankylosing spondylitis functional index; BASRI: Bath ankylosing spondylitis radiology index; CBir1: anti-flagellin; CSMC: Cedars-Sinai Medical Center; ELISA: enzyme-linked immunosorbent assay; fCAL: fecal calprotectin; HLA: human leukocyte antigen; IBD: inflammatory bowel disease; IL: interleukin; mAb: monoclonal antibody; NSAID: nonsteroidal anti-inflammatory drug; OmpC: anti-*Escherichia coli *outer membrane porin C; Th: T-helper type; TNF: tumor necrosis factor.

## Competing interests

The authors declare that they have no competing interests.

## Authors' contributions

FGM designed the project, collected data, analyzed data, and wrote the manuscript draft. SRT designed the project, analyzed data, and edited the manuscript. DB designed the analytic plan and provided statistical support and analysis consultancy. CJL analyzed serologic data. DPBM designed the project, analyzed data, and edited the manuscript. MHW designed the study, recruited research subjects, supervised data collection, analyzed data, and edited the manuscript. JDR helped design the project and helped analyze data. All authors read and approved the final manuscript.
